# HIV and STI Testing Preferences for Men Who Have Sex with Men in High-Income Countries: A Scoping Review

**DOI:** 10.3390/ijerph19053002

**Published:** 2022-03-04

**Authors:** Varsicka Kularadhan, Joscelyn Gan, Eric P. F. Chow, Christopher K. Fairley, Jason J. Ong

**Affiliations:** 1 School of Rural Health, Monash University, Bendigo, VIC 3550, Australia; 2 Melbourne Medical School, The University of Melbourne, Parkville, VIC 3010, Australia; joscelyng@student.unimelb.edu.au; 3 Central Clinical School, Monash University, Melbourne, VIC 3800, Australia; echow@mshc.org.au (E.P.F.C.); cfairley@mshc.org.au (C.K.F.); jason.ong@monash.edu (J.J.O.); 4 Melbourne Sexual Health Centre, Alfred Health, Carlton, VIC 3053, Australia; 5 Melbourne School of Population and Global Health, The University of Melbourne, Parkville, VIC 3010, Australia; 6 Department of Clinical Research, London School of Hygiene and Tropical Medicine, London WC1E 7HT, UK

**Keywords:** HIV, sexually transmitted infection, health service delivery, men who have sex with men

## Abstract

**Background:** Regular testing for HIV and other sexually transmitted infections (STI) is recommended at least annually for sexually active men who have sex with men (MSM) in most high-income countries. To encourage regular use of HIV and STI testing and treatment services for MSM, we reviewed the literature to summarise the attributes of an HIV/STI testing service that MSM prefer. **Method:** We conducted a scoping review, searching PubMed, EMBASE, PsycINFO and CINAHL in January 2020 for articles reporting primary data on the preferences of MSM (living in high-income countries) for HIV/STI testing services. Two reviewers independently screened titles and abstracts and any discrepancies were resolved by a third reviewer. We extracted data on the service attributes that MSM preferred and summarised these thematically using a socioecological framework. **Results:** In total, 1464 publications were identified, 220 full texts were read and 57 were included in the final analysis. We found 21 articles addressing ‘individual’ attributes, 50 articles addressing ‘service’ attributes and 17 articles addressing ‘societal’ attributes. The key themes of preferences for HIV/STI testing services were: (1) the appeal of self-testing due to convenience and privacy; (2) the need to provide a variety of testing options; and (3) the influence of the testing experience, including confidentiality and privacy, tester characteristics and stigma. There were distinct patterns of preferences for subpopulations of MSM across studies, such as the preference of self-testing for young MSM, and of in-clinic testing for those who perceived themselves as high risk (i.e., with symptoms of STIs or exposed to a partner living with HIV). **Conclusion:** To make HIV/STI testing more accessible for MSM and encourage regular screening, it is important to address ‘individual’, ‘service’ and ‘societal’ attributes, such as enhancing the convenience of testing through self-testing, and providing a service that men feel comfortable and safe accessing. Furthermore, services should accommodate the preferences of diverse sub-populations within the MSM community.

## 1. Introduction

Worldwide, there was estimated to be 1.7 million new HIV infections in 2019, and although this has declined significantly over the last ten years, numerous countries are not on track to meet 2020 and 2030 global targets for reductions in HIV incidence and mortality [1,2]. Men who have sex with men (MSM) continue to be disproportionately affected by HIV and sexually transmitted infections (STIs), even in high-income countries [3]. Globally, MSM are 26 times more at risk of acquiring HIV compared to heterosexual men [4]. 

As early HIV and most STIs can be asymptomatic, timely access to testing and treatment is critical for controlling HIV/STIs [5]. Although screening frequency varies across countries, the current overall screening frequency is not enough to control the rising epidemics of STIs. Rates of STIs in various high-income countries, including the US, across Europe and Australia, continue to rise within the MSM population, with the incidence greater than that of women and men who have sex with women only [6,7,8]. HIV/STI testing amongst this population has been reported to be suboptimal. A US cross-sectional study found that one-third of the sample had not been tested in the previous two years. In Australia, low frequency of testing and incomplete testing (i.e., chlamydia and gonorrhoea testing at three body sites and syphilis testing alongside all HIV testing) in MSM have been identified as one of the main barriers to HIV/STI control [9,10]. In other high-income countries, such as the US and Western Europe, limited access to HIV testing and care, and financial barriers act as significant barriers for regular testing among MSM [1]. 

HIV/STI testing can be delivered in a variety of ways including via hospitals, general practice, community-based organisations and home testing. Over the last five years, there have been a growing body of studies reporting the various attributes of HIV/STI testing services that MSM prefer. To date, systematic reviews have only focused on preferences related to HIV self-testing, but there has not been a review that synthesises the attributes of testing services that MSM prefer [11,12]. We reviewed the literature to provide an overview of the attributes of an HIV/STI testing service that MSM prefer, and to identify barriers to access. This review may be utilised to adapt current services or create new services that account for these preferred attributes, in order to improve the uptake of HIV/STI screening among MSM and decrease the prevalence of HIV/STIs.

## 2. Materials and Methods

### 2.1. The Search Strategy and Inclusion Criteria

We conducted a systemic review (Prospero: CRD42020179720) of the existing literature on the attributes of HIV/STI testing services preferred by MSM in high-income countries. We aimed to answer the following review question “In MSM living in high-income countries, which attributes of HIV/STI testing services do they prefer and not prefer?” To allow comparability between articles, we did not include publications from low- and middle-income countries as there are significant differences in their health systems compared to high-income countries. A literature search was conducted in January 2020 and updated in February 2022 using four electronic databases: MEDLINE (via PubMed), EMBASE, PsycINFO and CINAHL. Additional literature was included from hand searches in the reference lists of included articles. The search terms included across all databases related to (1) sexually transmitted infections, (2) health services and (3) testing or patient preferences. The search terms were: (“sexually transmitted infection” OR “sexually transmitted disease” OR “STI” OR “STD”) AND (“health service” OR “sexual health service”) AND (“testing” OR “preference” OR “patient preference” OR “perspective” OR “acceptability” OR “experience” OR “satisfaction”). No search filters were used. The full search strategy is provided in Tables S1–S4. The initial search was screened independently for relevant articles based on titles and abstracts meeting the inclusion criteria by two authors (JG, VK). Any discrepancies were resolved by a third author (JO). 

To be included, studies were required to report primary data on the preferences for attributes of HIV/STI testing services among MSM living in high-income countries (as per the World Bank definition). Articles that were not written in English, were conducted in low- or middle-income countries or were dated before 2000 were excluded. All citations were imported onto the citations manager, EndNote X9, and duplicates were removed initially by the citation manager. Duplications found later on in the process were manually deleted. 

### 2.2. Data Analysis

Data on testing preferences of MSM were extracted independently by two authors (JG, VK) using standardised extraction forms. We extracted data related to the setting of the study, the attributes tested, whether attributes were preferred or not preferred, and the characteristics of the study population. Data from the extraction forms were coded thematically into predefined themes using a socioecological framework, a commonly used model in public health interventions to understand the influences of individual-, service- and societal-level factors [13]. Two reviewers (VK, JG) independently coded the data, with a third reviewer (JO) resolving any discrepancies. Key attributes were then identified from the coded data and expanded upon further. Our findings are reported according to the PRISMA (Preferred Reporting Items for Systematic review and Meta-Analysis) checklist (Table S5). 

## 3. Results

**Figure 1:**
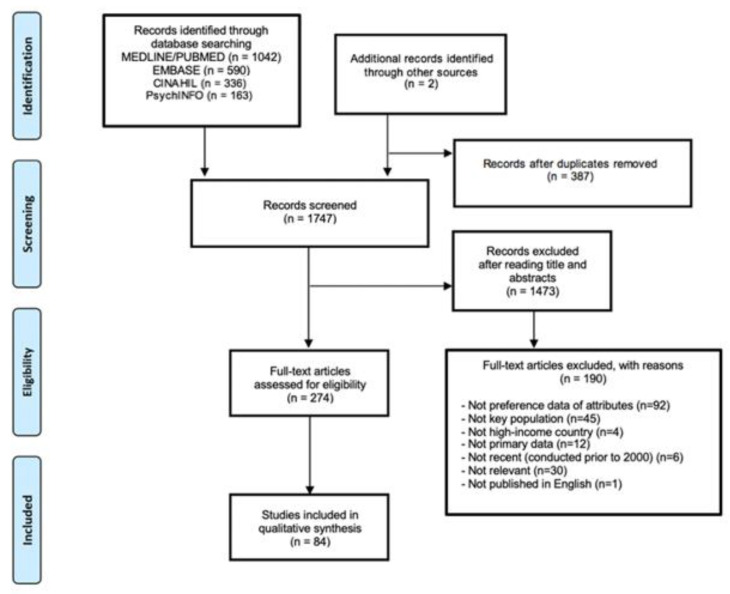
PRISMA flow diagram.

The initial search resulted in 1834 potentially relevant articles. An updated search resulted in 330 potentially relevant articles. After screening, 84 articles were included (Figure 1).

### 3.1. Study Characteristics

The majority of studies were conducted in the United States (US, *n* = 35), the United Kingdom (UK, *n* = 16), Australia (*n* = 14) and Europe (*n* = 11). A small number of studies were conducted in Canada (*n* = 6) and Asia (*n* = 2). Of 84 studies, 33 utilised interviews and focus groups, 30 utilised quantitative surveys, 9 were mixed methods, 7 were randomised controlled trials, 3 used data from records of pre-existing clinical services and 2 were discrete-choice experiments (Table S6). Forty-six were based on HIV testing, twenty on STI testing and eighteen on both HIV and STI testing. Eight studies focused on young MSM, five of which were specifically young African American MSM. Eight studies focused on MSM of colour. There were few data on first-time testers (*n* = 2) and MSM with high-risk profiles (*n* = 4). 

### 3.2. Socioecological Framework

**Figure 2:**
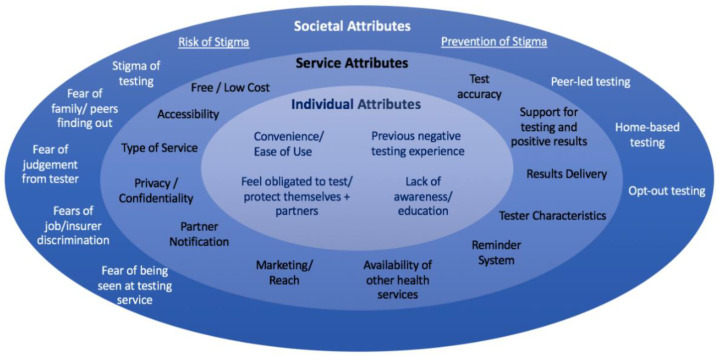
Summary of attributes using the socioecological framework.

Included articles were categorised using a socioecological framework: 30 articles addressed ‘individual’ attributes, 65 articles addressed ‘service’ attributes and 18 articles addressed ‘societal’ attributes. Figure 2 presents an overview of the attributes identified, and Tables S1–S3 categorise each study according to evidence of whether the attribute increases or decreases uptake of HIV/STI testing services. 

### 3.3. Individual Attributes

#### 3.3.1. Convenience

Convenience was a key attribute for MSM who preferred home self-sampling [14,15] or self-testing [16,17,18,19,20,21,22,23,24,25,26,27] (Table 1). A study conducted in the UK used focus groups and interviews with 44 MSM to explore the acceptability of home self-sampling kits [14]. The kits were highly acceptable, as men could perform the test at any time without needing to book an appointment and waiting times were important factors. It was also noted that these kits increased access to testing for participants who were geographically isolated or unable to access clinics due to work shift patterns [14]. Two studies conducted in the UK highlighted a preference for postal delivery of self-testing and self-sampling kits to increase convenience [15,20]. However, participants in one of these studies based on semi-structured interviews with 24 MSM about self-sampling raised concerns about the unreliability of postal services and the possibility of damage to specimens in transit [15]. This study also found that clinic attendance was preferred if participants were symptomatic, had been exposed to an infection, or a sexual partner had tested positive [15]. The other study, which conducted focus groups with 47 MSM about self-testing, found it was essential to offer a range of access options to maintain convenience and privacy [20]. 

Despite the appeal of the convenience of self-testing, we found three studies that identified issues with self-testing [28,29,30]. One study, conducted in the US, used a series of focus-group discussions with 21 young MSM of colour (aged 18–35 years) to explore the acceptability, preferences and usability of HIV self-test kits [29]. Identified issues included the instructions being too complicated and not user-friendly, privacy concerns with purchasing the kit and that the packaging itself was too bulky, clinical and outdated [29]. Another study, conducted in the UK, used semi-structured interviews to understand the acceptability of HIV self-testing (HIVST) amongst 37 MSM [30]. It identified difficulties with using the lancet; however, participants felt that this was only an issue during first-time use. Participants receiving repeat HIVST kits confirmed that repeated use increased confidence and competence [30]. Both studies discussed concerns about the user’s capacity to perform the test, as well as concerns about the accuracy and reliability of the test [29,30].

**Table 1:** Identified attributes of STI testing services—individual attributes.

Attribute	Attribute Examples
Convenience/Ease of Use of Testing	Self-testing (+) [16,17,18,19,20,21,22,23,24,25,26,27]
	Self-testing (−) [28,29,30]
	Self-sampling (+) [14,15,21]
Barriers	Previous negative experience (−) [28,31,32,33,34,35]
	Lack of awareness/education (−) [14,36,37]
	Confidentiality concerns in community-based settings (−) [38]
	Perceived low risk (−) [36,39]
	Lack of priority/lifestyle too busy (−) [33,39]
	Fear of positive result (−) [34]
	Medical mistrust (−) [35]
Individual Attitudes/Perceptions	Lack of testing among peers (−) [40]
	Feel obligated to test/protect themselves + partners (+) [14,15,19,22,26,40,41]
	Testing in response to risk incidents, unexpected symptoms or part of a sexual health routine (+) [42]

(+) study reported this as a preferred attribute; (−) study reported this as a non-preferred attribute.

#### 3.3.2. Previous Testing Experience

Six studies suggested a previous negative experience of the testing process to be an important barrier to HIV/STI testing [28,31,32,33,34,35]. Common themes which emerged included feeling embarrassed during previous testing experiences and discomfort about discussing their sexual history. A study using 30 semi-structured interviews with young African American MSM and transgender women in New York City (NYC) found that many had past experiences of testing filled with anxiety, which deterred them from future testing. In particular, negative relationships with and perceived negative attitudes of testers towards participants were important aspects of past testing [34]. Additionally, another study found that, even with positive past testing experiences, some men preferred not to go back to the location they first received their HIV diagnosis due to a now-negative association with the clinic [40]. This study, conducted in Amsterdam, used semi-structured qualitative interviews with 30 HIV-positive MSM to look at their sexual health practices in the year following their HIV diagnosis [40].

#### 3.3.3. Attitudes and Perceptions

Six studies identified that a feeling of obligation to protect themselves and their partners was an important reason for testing amongst MSM [14,15,19,22,39,40]. This finding was consistent across a variety of settings. A study from the US surveyed 460 participants at a needle exchange, three sex venues for MSM and an STI clinic [19]. Of the data from the sex venues in which the majority were MSM (87%, *n* = 139), most participants (84.4%) reported that not wanting to infect others was a strong motivator for HIV testing. Concern about exposure (78.9%) and wanting early treatment (78.2%) were also found to be significant motivators [19]. Another study which interviewed HIV-positive MSM in Amsterdam found that important motivators for regular STI testing were looking after their sexual health, protecting their partners and feeling more vulnerable to STIs (than before their diagnosis) [40]. 

A study which surveyed MSM with low intentions of actively seeking HIV testing in Spain found that those a majority of these men (49%) did not seek testing due to believing they were at low risk of contracting HIV compared to those who had high testing intentions [39].

### 3.4. Service Attributes

#### 3.4.1. Type of Service

Thirty-three studies identified home-based self-testing or self-sampling as the preferred method of testing [14,15,16,17,18,19,20,22,23,24,26,29,31,43,44,45,46,47,48,49,50,51,52,53,54,55,56,57,58,59,60,61] (Table 2). Three randomised controlled trials (RCT) found that HIVST increased testing frequency, and therefore was preferred by MSM compared to usual testing [17,21,54]. A US trial randomised 230 high-risk HIV-negative MSM to access oral fluid HIV self-tests at no cost versus testing as usual for 15 months [17]. There was an average use of 3.9 self-tests per person in the free self-testing group over the 15-month period, which was an increase of 1.7 tests per person compared to the testing as usual group [17]. Another US RCT randomised 65 HIV-negative MSM into three groups: HIVST kits by mail with a follow-up call from a counsellor (eTEST), standard HIVST kits with no follow-up (standard), or letters containing information about HIV testing locations (control) [53]. Delivery of HIVST kits by post at 3-month intervals increased testing, with all participants from the eTEST and standard groups testing at least once during the 7-month period compared to 72% of the control group. There was also nearly double the rate of repeat testing amongst these intervention groups [53]. The last RCT, conducted in Australia, randomised 362 men to free HIV self-testing plus facility-based testing, or facility-based testing only. Compared to standard care, self-testing doubled the frequency of testing in MSM and increased testing by nearly four times in non-recent testers [21]. 

Confidentiality or privacy was identified to be a common reason for preferring self-testing amongst MSM [14,25,27,34,49,51,52,60]. One study, conducted in the Netherlands, used semi-structured interviews to explore key factors which allowed for the successful implementation of social network testing with HIV self-tests [48]. It found that MSM valued not having to discuss their sexual identity or disclose sexual behaviours and avoided being seen at a testing facility [48]. Another study, which conducted 30 in-depth interviews with young MSM and transgender women in NYC, found similar themes; however, it also noted that living with parents may act as a barrier to self-testing, as they would like to avoid uncomfortable questions or conversations regarding testing [34]. 

A cross-sectional survey that recruited 15,704 MSM in England found that although genitourinary medicine (GUM) clinics were most popular amongst MSM, self-testing was preferred amongst first-time testers [52]. It also identified that this population valued the availability of a variety of testing methods and settings [52]. Another study that used semi-structured interviews with 24 MSM in the UK to explore their opinions on self-sampling kits identified that although the convenience of these kits was appreciated, testing at a clinic was preferred if they were symptomatic, exposed to an infection, or a sexual partner had tested positive [15].

One study conducted focus groups with 36 young African American MSM in Alabama, US, found overall negative opinions about self-testing at home due to concerns about accuracy and preferred to be tested by trusted physicians [28].

Another US study, which assessed data from a pilot RCT looking at STI self-collection, found that whilst self-testing gave MSM more control over their sexual health, there were some frustrations with tests which required blood-sample collection [21].

#### 3.4.2. Type of Testing

A study conducted in California, US, which used the surveys of 354 MSM clients of public testing services, identified the method of testing to be the least important attribute of HIV testing [18]. It found that accuracy, timeliness, the privacy of test disclosure, and linking of test results were equally ranked as the most important attributes. The availability of in-person counselling was ranked next and was also identified to be the strongest predictor of ‘loyalty’ to public clinic tests (even if other options were offered for free) [18]. Overall, there were widespread preferences for the type of testing (e.g., oral, blood, urine, rectal). A study conducted in NYC examined the computer-assisted self-interviews of 83 MSM to determine their preference of oral swab vs. blood-based HIV rapid testing [16]. It found that the majority preferred oral swabs, but they were more likely to consider the blood-based testing if it was cheaper, gave faster results and included co-testing of other STIs [16]. Similar results were also noted among a rural cohort of MSM in the US, which found if two HIV self-tests were available over the counter and cost the same, MSM were more likely to use the gym swab over the fingerpick blood test. However, if the cost of the fingerpick self-test cost half the price, and tested for other STIs, the preference for the fingerpick self-test increased [27]. Three studies discussed that the perceived greater accuracy of testing a blood sample compared to other samples was an important consideration in their preferences [20,28,34]. One study addressed the importance of wanting control over specimen collection, i.e., collecting their own specimen [50]. This study, conducted in Canada, used in-depth semi-structured interviews to explore the experiences of MSM who used an internet-based HIV and STI testing service, GetCheckedOnline.com [50].

#### 3.4.3. Rapid Testing

Eleven studies identified a preference for rapid testing [18,19,30,31,34,36,45,49,51,63,64]. Two studies, both conducted in Australia, highlighted that clients would test more frequently if rapid testing was available (rapid testing was not available in Australia at the time these studies were conducted) [51,63]. One of these studies, which used acceptability questionnaires to explore the HIV testing preferences of 1061 MSM, found that they preferred rapid testing as it was more convenient, more comfortable and less stressful than conventional HIV testing [63]. The other study, which used anonymous questionnaires to explore the syphilis testing preferences of 183 MSM, identified that 79% preferred rapid tests if they were available at clinics, and 70% of participants would test more frequently if this was the case [51]. The most common reasons for this were immediate results, reduced pain/invasiveness compared to venepuncture, and the convenience of not requiring a second appointment for results [51]. Another study, which used surveys with 460 MSM to identify motivators, barriers and preferences for HIV testing, found a preference for rapid testing, both in the clinic (27%) and at home (20%) [19]. Rapid testing was preferred due to faster results and less anxiety, with home-based rapid testing in particular allowing for more privacy and convenience. Only 31% of participants raised concerns about home-self testing; the majority were unsure about the accuracy of these tests, and some were concerned about user error and lack of counselling [19].

#### 3.4.4. Cost

Eight studies identified that free/low-cost testing was an important consideration for MSM [17,18,26,42,50,55,56,71]. A mixed-method study exploring the acceptability of self-testing in the UK used data collected from self-completed questionnaires and oral fluid specimen collections of 999 MSM and conducted 12 expert focus groups with MSM, health professionals, community organisations, entrepreneurs and activists [49]. It found that cost was an important consideration when using self-testing kits, with 80% of participants being likely to use self-tests if they were provided for free, compared to only 45.2% of MSM willing to pay for the tests [49]. An RCT conducted in Washington, US, randomly assigned 230 MSM to access free oral fluid HIV self-tests versus testing as usual [17]. Of these MSM, 15% would pay USD 40 or more for a self-test, 27% would pay USD 20–40, 33% would pay USD 10–20, 13% would pay less than USD 10, and 12% would only use a self-test if it were free. MSM also reported that the frequency of self-testing would be dependent on cost, with 87% saying they would test four or more times per year if it cost USD 5 compared to only 23% if it cost USD 50 [17]. Similarly, another US study found three-quarters of participants were willing to spend up to USD 20 for a HIV self-testing kit. However, many would rather utilise free community testing over paying for a HIV self-testing kit [26].

An online discrete choice experiment conducted in the UK with 620 MSM found that the majority had a preference for face-to-face testing with a healthcare professional compared to remote testing [68]. However, when the choice was between free remote testing or paying GBP 30 (~USD 40), there was a shift to remote testing [69]. Another study, which surveyed MSM clients of public testing services in California, US, found that if all tests were offered at no cost, although a public clinic test remained most preferred, self-tests became more popular [18].

A study that conducted focus groups with 36 young African American MSM in Alabama, US, found that privacy and confidentiality were important when seeking STI testing [28]. Participants highlighted that a private doctor’s office was ideal for these reasons, as there was no assumption of being there for STI testing, and medical professionals were obligated to maintain confidentiality. However, they also noted that this is not an option for many individuals as it requires private health insurance or the ability to pay out-of-pocket. Participants agreed that although STI testing at the local health department had a major drawback in terms of privacy and confidentiality, the low/no cost of testing and the availability of immediate treatment was considered more important [28].

#### 3.4.5. Tester Characteristics

Tester characteristics was an important consideration, particularly tester credibility/legitimacy [18,28,32,35,49], attitude [28,32,33,42,71,72], skill/knowledge [18,32,34,37,71,72] and familiarity with tester [18,28,29,36]. Gender of tester was not identified as an important consideration in any studies. 

A study of eight focus group discussions with 61 MSM in the UK explored their views and experiences of accessing sexual health services [32]. It identified that testers being professional, knowledgeable and non-judgemental was very important to this population. Participants noted that the staff’s demeanour and behaviour mades a significant difference in their testing experience, and they were also highly sensitive to how they felt the staff perceived them. They also noted that they would like staff to be well-informed about the latest research and practice evidence-based medicine. Some participants preferred services which specialised in working with MSM, stating that they felt more understood; however, this was not a collective preference [32]. Another study, conducted in California, US, used the surveys of 354 MSM clients of public testing services to identify the most important aspects of HIV testing [18]. Of the participants who preferred public clinic testing as their chosen type of service, 45% mentioned familiarity and professionalism of staff when asked about their reasoning [18].

Two studies conducted in Australia used sentinel surveillance, surveys and focus groups to evaluate PRONTO!, a peer-led, rapid testing, community shop-front model of HIV and STI testing [64,72]. Analysis of evaluation surveys from 416 MSM found that most participants were comfortable waiting for their results with peer-test facilitators and that they found these facilitators to be competent, professional and capable of providing any information and referrals required. The majority (65.3%) of participants indicated that they preferred testing with peer-test facilitators compared to sexual health doctors or nurses. The focus groups revealed that participants thought that one of the main benefits of the peer-testing model was that it allowed for meaningful discussion of sexual health and broader issues relevant to the population. They felt they could ask questions they would normally not ask other health professionals. Participants also valued the relationships built with peer staff and noted that the testing experience was enhanced by having testers they could relate to. Although confidentiality concerns were raised and some unease about potentially knowing the staff, this was outweighed by the professionalism displayed by testers [64,72]. 

#### 3.4.6. Results and Support

Six studies identified the importance of receiving positive results in person or over the phone rather than by text or another format [15,19,20,31,37,78]. A study conducted in the UK that focused on home-sampling kits highlighted that MSM found a ‘no news is good news’ approach caused anxiety and uncertainty, and participants preferred to be notified with a negative result [15]. It also found that they preferred to be offered various options for results delivery (i.e., phone, text, email, post) [15]. 

Eight studies identified the importance of education and counselling with HIV/STI testing [18,36,48,59,71,76,77,78], with seven studies identifying the lack of these services in self-testing being a barrier to testing [14,20,24,50,60,81,82]. A study that used data from the 2011/2012 Sex Now Survey (a serial online survey of MSM in Canada) to explore perceived benefits and drawbacks of Internet-based testing found that the biggest concerns regarding self-testing were the inability to see a health professional or to discuss results, and receiving results online [75]. Another study conducted six focus-group discussions with 47 MSM in England to examine their ideal HIVST service [20]. Participants had positive views of self-testing; however, they feared having no support if there was a positive result. A 24/7 telephone helpline was the most preferred method of support alongside HIVST [20].

### 3.5. Societal Attributes 

#### Stigma Associated with HIV/STI Testing

Ten studies identified stigma of HIV/STI testing to be a deterrent to testing (Table 3). A study conducted in the UK utilised twelve focus groups with 55 multi-professional, patient and provider ‘expert’ participants to explore barriers and facilitators of self-testing amongst MSM [49]. It was discussed that stigma associated with testing was a barrier to accessing sexual health services, and many MSM would not attend GUM clinics for testing for this reason. It was noted that self-testing would reduce this barrier and potentially reach MSM who were not testing due to this [49]. Another study conducted in-depth, semi-structured interviews with 16 MSM in Australia and explored barriers to accessing HIV and STI testing [37]. Stigma was highlighted to be a significant barrier within this community, including internalised stigma and fears of being publicly outed, with one participant highlighting the “culture of avoidance” amongst this population. Education about sexual minorities was suggested to be an important step in facing this issue [37]. Four of these studies [32,83], noted concerns about being seen at the clinic and its associated stigma. A study conducted eight focus-group discussions with 61 MSM in the UK, to explore their views and experiences of accessing sexual health services [32]. Many participants discussed being self-conscious attending a service and felt as though they were drawing attention to themselves and why they were there, with one participant describing it as a “walk of shame” [32]. Five studies suggested possible solutions to minimise the stigma surrounding HIV/STI testing. These included the implementation of peer-led testing, home self-testing and an opt-out testing system [14,30,36,62,76].

## 4. Discussion

This scoping review identified attributes of HIV/STI testing services that influence MSM to access regular testing. Synthesising the extant literature provides an overview of the most important attributes, which are crucial in optimising current testing options for this population. We found that most studies focused on ‘service-level’ attributes, highlighting the appeal of self-testing, rapid-testing, free/cheap services and skilled testers with positive attitudes. The importance of convenience, offering various testing options and providing a positive testing experience were key themes throughout this review. These data provide valuable insights to assist in adapting current service models or creating new services focused on improving access to testing for MSM. 

Access to self-testing was an important attribute across various studies; this was primarily due to convenience. This was reflected by the increased uptake of testing found in three RCTs [17,21,54]. Although concerns were raised about the accuracy of self-testing and the difficulty of use, it remained one of the most popular options across studies. Services should evaluate how to best utilise self-testing to encourage regular testing amongst MSM. Making self-testing readily available and improving promotion would likely be beneficial and should be explored. 

For service-level attributes, a range of preferences were identified such as the method of testing and results delivery. It was noted that services should ideally offer a variety of options to appeal to subpopulations of MSM. Cost was a key component, with cheaper or free options having a significant impact on the services MSM would access and how regularly they get tested [17,18,42,50,55,56,71]. Identifying the type of testing most suitable to individuals by taking these preferences into consideration early on would likely greatly impact MSM returning for regular testing. Additionally, appropriate education was needed for HIV/STI testing, with some preferences based on incomplete information. For example, perceived greater accuracy of blood testing drove a preference for this method of testing [20,28,34]. Overall, the data suggest that in combination with appropriate pre-testing counselling, a service that offers both self-testing and standard testing (at least one of which is free or cheap) and the ability to choose how results are delivered would be popular amongst MSM. Additionally, education surrounding the options available for testing should be provided on a larger scale, both within appointments and across other platforms such as social media. 

The testing experience was an important influence on MSM and their HIV/STI testing habits. This included confidentiality and privacy, tester characteristics and stigma, which were particularly important considerations amongst this population. Confidentiality and privacy were recurring themes throughout study findings and greatly influenced preferences. It was identified as one of the key reasons self-testing was preferred, and was a barrier for accessing in-clinic testing. A previous negative testing experience was a significant barrier to testing in the future [28,31,32,33,34,35]. The barrier that stigma imposes on HIV/STI testing can only be partially addressed by optimising testing services as stigma is a community-wide issue that will require interventions directed towards the entire community. However, assessing the benefits and feasibility of implementing strategies such as peer-led testing, home self-testing, and an opt-out testing system is an important consideration for all testing services [14,30,64,72,84].

Distinct patterns of preferences for subpopulations of MSM were identified across several studies. Self-testing appeared to be more attractive to young MSM and first-time testers, while in-clinic testing was preferred by those who perceived themselves as high risk (i.e., symptomatic or exposed to a partner with known infection) [15]. Furthermore, African American MSM generally preferred to be tested by health professionals and to receive information about testing and their test results from clinicians [28,81,82].

The strength of this scoping review is that, to our knowledge, this is the first attempt to synthesise the data of the attributes of HIV/STI testing preferred by MSM living in high-income countries. Limitations of this study include that the majority of studies were conducted within the US, the UK and Australia. Therefore, data from other high-income countries, particularly Asian and European countries, were limited. We only included studies published in English and thus potentially missed relevant studies from high-income countries. We also did not include grey literature which may contain other relevant data. Additionally, most of the studies analysed focused on comparing specific attributes; for example, preferences of a select number of testing methods, rather than looking at how people traded off between numerous attributes and their relative importance. We only found one study using a discrete-choice experiment that attempted this [69]. 

Further research is required into the preferences of subpopulations of MSM. It was noted that the few studies that addressed subpopulations such as African American MSM might have skewed the results of this scoping review, and therefore in the future, research focusing solely on these subpopulations is required. Additionally, further research should be conducted on testing preferences for MSM with different risk profiles, or symptomatic and asymptomatic MSM, to identify the most effective approach to testing. This scoping review also only included high-income countries, and it would be beneficial to also look at data from low- and middle- income countries. 

## 5. Conclusions

This scoping review found that MSM have a diverse range of preferences for HIV/STI testing services, and it is important to address ‘individual’, ‘service’ and ‘societal’ attributes in order to make HIV/STI testing more accessible and encourage regular screening. This includes enhancing the convenience of testing and providing a service that men feel comfortable and safe accessing. Self-testing is a valuable tool to increase access to testing amongst this population; however, offering variety within a service is equally essential to enhance reach. Services should accommodate the preferences of diverse sub-populations within the MSM community, and further research is required to facilitate this.

## Figures and Tables

**Table 2:** Identified attributes of STI testing services—Service Attributes.

Attribute	Attribute Examples
Type of Service	Self-testing/Self-sampling (+) [14,15,16,17,18,19,20,22,23,24,26,29,31,43,44,45,46,47,48,49,50,51,52,53,54,55,56,57,58,59,60,61]
	Self-collection (+) [21,31]
	Self-collection (−) [18,21]
	Mobile testing (+) [62]
	Mobile testing (−) [32]
	Online testing service (+) [43]
	Rapid testing (+) [18,19,30,31,34,36,45,49,51,63,64]
	Express service (+) [65]
Accessibility	Appointment system availability (−) [28]
	Appointment system ease of use (+) [66]
	Walk-in Service (+) [62]
	Waiting times (−) [66]
	Self-testing kits available at a variety of locations (+) [22,54,59]
	Non-specialist setting (−) [67]
Type of Test	Oral (+) [16]
	Rectal (+) [68]
	Urine (+) [28]
	Blood (+) [25,34]
	Venepuncture (−) [19]
	Other attributes (e.g., cost, speed) > type of test (+) [16,27]
Accuracy	High accuracy (+) [26,34]
	Accuracy > convenience of sample collection (+) [14]
	Concerns about accuracy/reliability of self-testing and rapid testing (−) [22,28]
Cost	Free/low cost (+) [17,18,27,49,54,55,69,70]
	No health insurance (−) [28]
	Cost (−) [27,37,59]
Privacy, Confidentiality & Anonymity	Fears of disclosing sexual identity or behaviour in an unfamiliar environment (−) [48]
	Privacy/anonymity when testing + receiving results (+) [28]
	Open waiting room (−) [14]
	Non-specialist setting (−) [32]
	Picking up of self-testing kit from pharmacy/clinic (−) [29]
	Named reporting (−) [19]
	Providing personal information online (−) [71]
Tester Characteristics	Credibility of tester and legitimacy (+) [18,28,32,35,48]
	Tester attitude (+) [28,32,33,42,71,72,73]
	Risk of being recognised (−) [64]
	Familiarity with tester/comfortable environment (+) [18,27,28,29,64]
	Skill/knowledge of tester (+) [18,32,34,37,71,72]
	Healthcare professional (+) [28]
	Peer-testing (+) [51]
	Lack of trans knowledge (−) [35]
Results Delivery	In person/via phone call if positive (+) [15,19,20,31,71,73,74]
	Online results/via phone app (+) [27,43,58,73]
	Online results (−) [59,75]
	Quick/immediate results (+) [27,59]
	Through text if negative (+) [31]
Support for Testing and Positive Results	Education/counselling (+) [18,36,48,59,71,76,77,78]
	Availability of immediate treatment (+) [27,28]
	Face to face counselling (−) [19]
	Linkage to care (+) [79]
	Linkage of results to other health professionals (+) [18,27,73]
	Home-based testing: support offered (+) [45]
	Partner delivered partner therapy (+) [55]
	Anonymous partner notification (+) [55]
	Self-testing: lack of support (−) [14,20,24,38,49,59,75]
Reminder System	From local health department (+) [28]
	From online service (+) [50]
	From STI testing service (+) [36]
Partner Notification	Via phone app (+) [43,80]
	Anonymous e-card (+) [70]
Availability of Other Health Services	Offering other health services (+) [36]
	Self-testing: lack of availability of other health services (−) [49]
	Lack of co-testing of other STIs (−) [64]
	Integrating testing with ongoing monitoring for hormone therapy (+) [35]
	Availability of condoms and lubricants (+) [36]
Reach/Marketing	Use of apps/internet (+) [70]

(+) study reported this as a preferred attribute; (−) study reported this as a non-preferred attribute.

**Table 3:** Identified attributes of STI testing services—societal attributes.

Attribute	Attribute Examples
Risk of Being Stigmatised	Fear of being seen/community finding out (−) [32,83]
	Fears of family/peers finding out (−) [15,83]
	Fears of job/insurer discrimination (−) [19]
	Stigma of STI testing (−) [19,28,32,34,37,41,48,49,71,83]
	Fear of judgement from tester/negative treatment (−) [38,66]
Preventing Stigma	Peer-led testing (+) [64,72]
	Home-self testing (+) [14,30]
	Opt-out testing (+) [84]

(+) study reported this as a preferred attribute; (−) study reported this as a non-preferred attribute.

## Data Availability

All relevant data have been published in the manuscript or in the supplementary material. Further details can be obtained by writing to the corresponding author.

## Supplementary Materials

The following supporting information can be downloaded at: https://www.mdpi.com/article/10.3390/ijerph19053002/s1: Table S1: Search Strategy: PUBMED; Table S2: Search Strategy: EMBASE; Table S3: Search Strategy: PsychINFO; Table S4: Search Strategy: CINAHL; Table S5: PRISMA Checklist; Table S6: Summary of Included Study Characteristics and PREFS* Scores.
