# Supplementary material for: HIV and STI Testing Preferences for Men Who Have Sex with Men in High-Income Countries: A Scoping Review
Source: Int J Environ Res Public Health. 2022 Mar 4;19(5):3002. doi: 10.3390/ijerph19053002 (PMC8910668; doi:10.3390/ijerph19053002)
Supplement: Supplementary file 1 [file ijerph-19-03002-s001.zip › ijerph-1569375-supplementary.pdf]

Optimizing HIV and STI testing services for men who have sex with men in high-income countries: A Systematic Review

Table S1. Search Strategy: PUBMED

Set	Search	Results
#1	(STD OR STI OR HIV OR sexual health OR genitourinary) AND (service* OR test* OR diagnosis OR management OR treatment OR care) AND (prefer* OR satisfaction OR acceptability OR perspective* OR perception* OR qualitative OR value* OR experience*)	155,079
#2	(((((sexually transmitted infection[MeSH Terms]) OR sexually transmitted disease[MeSH Terms]) AND (sexual health service[MeSH Terms]) OR health service)))	2,513,004
#3	(((((((sexually transmitted disease[MeSH Terms]) OR STI) OR STD) OR Sexually transmitted infection[MeSH Terms]))) AND (((((diagnosis[MeSH Terms]) OR service) OR Testing))) AND ((((((Perspective) OR Perception) OR acceptability) OR Preference) OR Satisfaction) OR experience))	7998
#4	(((((((sexually transmitted disease[MeSH Terms]) OR STI) OR STD) OR Sexually transmitted infection[MeSH Terms]))) AND (((((diagnosis[MeSH Terms]) OR service) OR Testing))) AND ((((Perspective) OR Perception) OR acceptability)))	342
#5	(((((sexually transmitted disease[MeSH Terms]) OR sexually transmitted infection[MeSH Terms]) OR STI) OR STD) AND Sexual health service[MeSH Terms]) AND internet[MeSH Terms]	342
#6	((((((sexually transmitted disease[mesh terms]) or std or sexually transmitted infection[mesh terms]) or sti and health service) and preference)) and testing)	216
#7	((((((sexually transmitted disease[mesh terms]) or std or sexually transmitted infection[mesh terms]) or sti and sexual health service) and preference)) and testing)	213
#8	((((sexually transmitted infection[MeSH Terms]) OR sexually transmitted disease[MeSH Terms]) AND patient preference[MeSH Terms]))	179
#9	((((((sexually transmitted infection[MeSH Terms]) OR sexually transmitted disease[MeSH Terms]) AND sexual health service[MeSH Terms]))))) AND preference	135
#10	((((((sexually transmitted disease[mesh terms]) or std or sexually transmitted infection[mesh terms]) or sti and sexual health service) and testing))) AND patient preference[MeSH Terms]	70
#11	(((((((sexually transmitted disease[MeSH Terms]) OR sexually transmitted infection[MeSH Terms]) OR STI) OR STD) AND Sexual health service[MeSH Terms]))) AND patient preference[MeSH Terms]	48

Table S2. **Search Strategy: EMBASE**

Set	Search	Results
#1	((STD or STI or HIV or sexual health or genitourinary) and (service* or test* or diagnosis or management or treatment or care) and (prefer* or satisfaction or acceptability or perspective* or perception* or qualit* or value* or experience*)).af.	93,747
#2	((Sexually transmitted infection or STI).kw. or STD.af. or Sexually transmitted disease.af.) and health service.af. and testing.af.	1154
#3	(STD OR STI OR HIV OR sexual health OR genitourinary) AND (service* OR test* OR diagnosis OR management OR treatment OR care) AND (prefer* OR satisfaction OR acceptability OR perspective* OR perception* OR qualit* OR value* OR experience*).kw.	804
#4	((Sexually transmitted infection or sexually transmitted disease).kw. or STI.af. or STD.af.) and testing.kw.	590
#5	((Sexually transmitted infection or sexually transmitted disease).kw. or STI.af. or STD.af.) and internet.kw.	84

Table S3. **Search Strategy: PsychINFO**

Set	Search	Results
#1	((STD or STI or HIV or sexual health or genitourinary) and (service* or test* or diagnosis or management or treatment or care) and (prefer* or satisfaction or acceptability or perspective* or perception* or qualit* or value* or experience*)).af.	98,305
#2	(Sexually transmitted infection.mh. or STI.af. or sexually transmitted disease.mh. or STD.af.) and (testing.af. or diagnosis.af. or service.mh.) and (preference.mh. or perspective.af. or perception.af. or acceptability.af. or Satisfaction.af. or experience.af.)	6186
#3	(((Sexually transmitted infection or sexually transmitted disease) and patient preferences).mh. and testing.af. and health service.mh.) or sexual health service.af.	163
#4	(Sexually transmitted infection.mh. or STI.af. or sexually transmitted disease.mh. or STD.af.) and patient preference.mh.	24
#5	(((Sexually transmitted infection or sexually transmitted disease) and patient preferences).mh. and testing.af. and health service.mh.) or sexual health service.af.	0
#6	(Sexually transmitted infection.mh. or STI.af. or sexually transmitted disease.mh. or STD.af.) and sexual health service.mh. and patient preference.af.	0

Table S4. **Search Strategy: CINAHL**

Set	Search	Results
#1	(STD OR STI OR HIV OR sexual health OR genitourinary) AND (service* OR test* OR diagnosis OR management OR treatment OR care) AND (prefer* OR satisfaction OR acceptability OR perspective* OR perception* OR qualit* OR value* OR experience*)	26,222
#2	sexually transmitted diseases OR MW sexually transmitted infections AND MW sexual health services AND MW patient preference AND MW testing	5421
#3	sexually transmitted diseases OR MW sexually transmitted infections AND MW sexual health services AND MW patient preference	5421
#4	(((((((sexually transmitted disease[MeSH Terms]) OR STI) OR STD) OR Sexually transmitted infection[MeSH Terms]))) AND (((((diagnosis[MeSH Terms]) OR service) OR Testing))) AND ((((((Perspective) OR Perception) OR acceptability) OR Preference) OR Satisfaction) OR experience))	336
#5	(sexually transmitted diseases or sexually transmitted infections or sti or std) AND MW patient preference AND MW sexual health services	0

Table S5. PRISMA Checklist

Section/topic	#	Checklist item	Reported on page #
**TITLE**			
Title	1	Identify the report as a systematic review, meta-analysis, or both.	1
**ABSTRACT**			
Structured summary	2	Provide a structured summary including, as applicable: background; objectives; data sources; study eligibility criteria, participants, and interventions; study appraisal and synthesis methods; results; limitations; conclusions and implications of key findings; systematic review registration number.	1-2
**INTRODUCTION**			
Rationale	3	Describe the rationale for the review in the context of what is already known.	3
Objectives	4	Provide an explicit statement of questions being addressed with reference to participants, interventions, comparisons, outcomes, and study design (PICOS).	3
**METHODS**			
Protocol and registration	5	Indicate if a review protocol exists, if and where it can be accessed (e.g., Web address), and, if available, provide registration information including registration number.	4
Eligibility criteria	6	Specify study characteristics (e.g., PICOS, length of follow-up) and report characteristics (e.g., years considered, language, publication status) used as criteria for eligibility, giving rationale.	4
Information sources	7	Describe all information sources (e.g., databases with dates of coverage, contact with study authors to identify additional studies) in the search and date last searched.	4
Search	8	Present full electronic search strategy for at least one database, including any limits used, such that it could be repeated.	4, Sup. 1
Study selection	9	State the process for selecting studies (i.e., screening, eligibility, included in systematic review, and, if applicable, included in the meta-analysis).	4
Data collection process	10	Describe method of data extraction from reports (e.g., piloted forms, independently, in duplicate) and any processes for obtaining and confirming data from investigators.	5
Data items	11	List and define all variables for which data were sought (e.g., PICOS, funding sources) and any assumptions and simplifications made.	5
Risk of bias in individual studies	12	Describe methods used for assessing risk of bias of individual studies (including specification of whether this was done at the study or outcome level), and how this information is to be used in any data synthesis.	5

Section/topic	#	Checklist item	Reported on page #
Summary measures	13	State the principal summary measures (e.g., risk ratio, difference in means).	5
Synthesis of results	14	Describe the methods of handling data and combining results of studies, if done, including measures of consistency (e.g., I^2) for each meta-analysis.	5
Risk of bias across studies	15	Specify any assessment of risk of bias that may affect the cumulative evidence (e.g., publication bias, selective reporting within studies).	5
Additional analyses	16	Describe methods of additional analyses (e.g., sensitivity or subgroup analyses, meta-regression), if done, indicating which were pre-specified.	n/a
**RESULTS**			
Study selection	17	Give numbers of studies screened, assessed for eligibility, and included in the review, with reasons for exclusions at each stage, ideally with a flow diagram.	6
Study characteristics	18	For each study, present characteristics for which data were extracted (e.g., study size, PICOS, follow-up period) and provide the citations.	6-7, Sup. 3
Risk of bias within studies	19	Present data on risk of bias of each study and, if available, any outcome level assessment (see item 12).	Sup. 3
Results of individual studies	20	For all outcomes considered (benefits or harms), present, for each study: (a) simple summary data for each intervention group (b) effect estimates and confidence intervals, ideally with a forest plot.	7-16
Synthesis of results	21	Present results of each meta-analysis done, including confidence intervals and measures of consistency.	20-24
Risk of bias across studies	22	Present results of any assessment of risk of bias across studies (see Item 15).	n/a
Additional analysis	23	Give results of additional analyses, if done (e.g., sensitivity or subgroup analyses, meta-regression [see Item 16]).	n/a
**DISCUSSION**			
Summary of evidence	24	Summarize the main findings including the strength of evidence for each main outcome; consider their relevance to key groups (e.g., healthcare providers, users, and policy makers).	16-18
Limitations	25	Discuss limitations at study and outcome level (e.g., risk of bias), and at review-level (e.g., incomplete retrieval of identified research, reporting bias).	18
Conclusions	26	Provide a general interpretation of the results in the context of other evidence, and implications for future research.	18-19
**FUNDING**			
Funding	27	Describe sources of funding for the systematic review and other support (e.g., supply of data); role of funders for the systematic review.	19

Table S6. Summary of Included Study Characteristics and PREFS* Scores

	Author	Year of Study	Country	Method	PREFS Score
4	Alarcon Gutierrez[1]	2015-2016	Spain	Interview	4
9	Balan[2]	Published 2016	USA	Interview	4
10	Balan[3]	2016-2017	USA	Interview	3
14	Barnard[4]	2016	UK	Data from pre-existing services	3
16	Bauermeister[5]	Published 2015	USA	Randomised controlled trial	4
18	Baytop[6]	2008-2010	USA	Data from pre-existing services	4
220	Biello[7]	2018 - 2019	USA	Randomised controlled trial	3
39	Chen[8]	2009	Australia	Questionnaire	4
40	Clark[9]	2012-2015	USA	Questionnaire	4
43	Cohall[10]	2005	USA	Questionnaire	4
48	Conway[11]	2011-2012	Australia	Questionnaire	3
222	Contesse[12]	2017	USA	Questionnaire	2
53	Cushman[13]	2014	USA	Questionnaire	3
223	D'Angelo[14]	2018 – 2019	USA	Questionnaire	3
54	Datta[15]	2015	UK	Focus group	2
58	den Daas[16]	2017	Netherlands	Interview	3
63	Dodge[17]	Published 2010	USA	Interview	3
65	Eaton[18]	2017-2018	USA	Focus group	3
69	Feinstein[19]	2013	USA	Interview	3
73	Fields[20]	2015 - 2016	USA	Interview	3
74	Flowers[21]	2014	UK	Mixed method	3
76	Frye[22]	2014	USA	Interview	3
81	Gilbert[23]	2011-2012	Canada	Questionnaire	4
83	Gilbert[24]	2015-2016	Canada	Questionnaire	4
90	Gu[25]	2006	Hong Kong	Interview	4
98	Heijman[26]	2008-2009	Amsterdam	Interview	3
105	Holt[27]	2006-2008	Australia	Interview	3

106	Hottes[28]	Published 2012	Canada	Focus group	3
109	Hoyos[29]	2008	Spain	Data from pre-existing services	3
225	Hoyos[30]	2016	Greece, Spain, France, Germany, Denmark	Questionnaire	3
227	Iribarren[31]	2014 – 2017	USA, Puerto Rico	Randomised controlled trial	4
233	Jamil[32]	2013-2015	Australia	Randomised controlled trial	4
118	Katz[33]	2010-2014	USA	Randomised controlled trial	4
123	Knight[34]	2015-2017	Canada	Interview	3
124	Knight[35]	2013-2014	Australia	Cross sectional analysis	4
125	Knussen[36]	2005	UK	Questionnaire	4
129	Lea[37]	2016	Australia	Interview	3
131	Lechuga[38]	Published 2013	USA	Questionnaire	4
132	Lee[39]	2009	Australia	Questionnaire	3
134	Leitinger[40]	2013-2015	Australia	Questionnaire	4
137	Llewellyn[41]	2006	UK	Focus groups	3
141	Martin[42]	Published 2012	Australia	Questionnaire	4
142	Martin[43]	2010	Australia	Questionnaire	4
144	Maxwell[44]	Published 2018	UK	Questionnaire	4
147	Medline[45]	Published 2017	USA	Focus group	3
152	Miners[46]	2017	UK	Discrete Choice Experiment	4
160	Pant Pai[47]	2016-2017	Canada	Questionnaire	4
169	Pollard[48]	2009	UK	Focus group	3
181	Ryan[49]	2013-2015	Australia	Mixed method	4
183	Scheim[50]	2013	Canada	Interview	3

189	Skolnik[51]	1999	USA	Questionnaire	3
193	Spielberg[52]	1998	USA	Questionnaire	4
196	Strömdahl[53] [60]	2013	Sweden	Questionnaire	4
198	Sun[54]	2013-2014	USA	Questionnaire	4
202	Tobin[55]	Published 2018	USA	Interview	3
207	Wayal[56]	2005-2007	UK	Interview	3
210	Witzel[57]	2017 - 2018	UK	Interview	3
211	Witzel[58]	2014	UK	Questionnaire	4
212	Witzel[59]	2015	UK	Focus group	3
213	Wohlfeiler[60]	2009-2010	UK	Questionnaire	4
216	Wray[61]	2016-17	USA	Randomised controlled trial	4

* PREFS - purpose, respondents, explanation, findings, significance

Reference List for Table S6

1. Alarcón Gutiérrez, M.; Fernández Quevedo, M.; Martín Valle, S.; Jacques-Aviñó, C.; Díez David, E.; Caylà, J.A.; García de Olalla, P. Acceptability and effectiveness of using mobile applications to promote HIV and other STI testing among men who have sex with men in Barcelona, Spain. *Sex Transm Infect* **2018**, *94*, 443-448, doi:10.1136/sextrans-2017-053348.
2. Balán, I.; Frasca, T.; Ibitoye, M.; Dolezal, C.; Carballo-Diéguez, A. Fingerprick Versus Oral Swab: Acceptability of Blood-Based Testing Increases If Other STIs Can Be Detected. *AIDS Behav* **2017**, *21*, 501-504, doi:10.1007/s10461-016-1497-4.
3. Balán, I.C.; Lopez-Rios, J.; Nayak, S.; Lentz, C.; Arumugam, S.; Kutner, B.; Dolezal, C.; Macar, O.U.; Pabari, T.; Wang Ying, A., et al. SMARTtest: A Smartphone App to Facilitate HIV and Syphilis Self- and Partner-Testing, Interpretation of Results, and Linkage to Care. *AIDS Behav* **2020**, *24*, 1560-1573, doi:10.1007/s10461-019-02718-y.
4. Barnard, S.; Free, C.; Bakolis, I.; Turner, K.M.E.; Looker, K.J.; Baraitser, P. Comparing the characteristics of users of an online service for STI self-sampling with clinic service users: a cross-sectional analysis. *Sex Transm Infect* **2018**, *94*, 377-383, doi:10.1136/sextrans-2017-053302.
5. Bauermeister, J.A.; Pingel, E.S.; Jadwin-Cakmak, L.; Harper, G.W.; Horvath, K.; Weiss, G.; Dittus, P. Acceptability and preliminary efficacy of a tailored online HIV/STI testing intervention for young men who have sex with men: the Get Connected! program. *AIDS Behav* **2015**, *19*, 1860-1874, doi:10.1007/s10461-015-1009-y.
6. Baytop, C.; Royal, S.; Hubbard McCree, D.; Simmons, R.; Tregerman, R.; Robinson, C.; Johnson, W.D.; McLaughlin, M.; Price, C. Comparison of strategies to increase HIV testing among African-American gay, bisexual, and other men who have sex with men in Washington, DC. *AIDS Care* **2014**, *26*, 608-612, doi:10.1080/09540121.2013.845280.
7. Biello, K.B.; Horvitz, C.; Mullin, S.; Mayer, K.H.; Scott, H.; Coleman, K.; Dormitzer, J.; Norelli, J.; Hightow-Weidman, L.; Sullivan, P., et al. HIV self-testing and STI self-collection via mobile apps: experiences from two pilot randomized controlled trials of young men who have sex with men. *Mhealth* **2021**, *7*, 26, doi:10.21037/mhealth-20-70.
8. Chen, M.Y.; Bilardi, J.E.; Lee, D.; Cummings, R.; Bush, M.; Fairley, C.K. Australian men who have sex with men prefer rapid oral HIV testing over conventional blood testing for HIV. *Int J STD AIDS* **2010**, *21*, 428-430, doi:10.1258/ijsa.2010.009552.
9. Clark, H.A.; Oraka, E.; DiNenno, E.A.; Wesolowski, L.G.; Chavez, P.R.; Pitasi, M.A.; Delaney, K.P. Men Who Have Sex with Men (MSM) Who Have Not Previously Tested for HIV: Results from the MSM

Testing Initiative, United States (2012-2015). *AIDS Behav* **2019**, *23*, 359-365, doi:10.1007/s10461-018-2266-3.
10. Cohall, A.; Dini, S.; Nye, A.; Dye, B.; Neu, N.; Hyden, C. HIV testing preferences among young men of color who have sex with men. *Am J Public Health* **2010**, *100*, 1961-1966, doi:10.2105/ajph.2008.140632.
11. Conway, D.P.; Guy, R.; Davies, S.C.; Couldwell, D.L.; McNulty, A.; Smith, D.E.; Keen, P.; Cunningham, P.; Holt, M. Rapid HIV Testing Is Highly Acceptable and Preferred among High-Risk Gay And Bisexual Men after Implementation in Sydney Sexual Health Clinics. *PLoS One* **2015**, *10*, e0123814, doi:10.1371/journal.pone.0123814.
12. Contesse, M.G.; Fredericksen, R.J.; Wohlfeiler, D.; Hecht, J.; Kachur, R.; Strona, F.V.; Katz, D.A. Acceptability of Using Geosocial Networking Applications for HIV/Sexually Transmitted Disease Partner Notification and Sexual Health Services. *Sex Transm Dis* **2020**, *47*, 41-47, doi:10.1097/olq.0000000000001089.
13. Cushman, T.A.; Graves, S.K.; Little, S.J. Attitudes and Preferences Regarding the Use of Rapid Self-Testing for Sexually Transmitted Infections and HIV in San Diego Area Men Who Have Sex With Men. *Open Forum Infect Dis* **2019**, *6*, ofz043, doi:10.1093/ofid/ofz043.
14. D'Angelo, A.B.; Morrison, C.A.; Lopez-Rios, J.; MacCrate, C.J.; Pantalone, D.W.; Stief, M.; Grov, C. Experiences Receiving HIV-Positive Results by Phone: Acceptability and Implications for Clinical and Behavioral Research. *AIDS Behav* **2021**, *25*, 709-720, doi:10.1007/s10461-020-03027-5.
15. Datta, J.; Reid, D.; Hughes, G.; Mercer, C.H.; Wayal, S.; Weatherburn, P. Places and people: the perceptions of men who have sex with men concerning STI testing: a qualitative study. *Sex Transm Infect* **2018**, *94*, 46-50, doi:10.1136/sextrans-2016-052983.
16. den Daas, C.; Geerken, M.B.R.; Bal, M.; de Wit, J.; Spijker, R.; Op de Coul, E.L.M. Reducing health disparities: key factors for successful implementation of social network testing with HIV self-tests among men who have sex with men with a non-western migration background in the Netherlands. *AIDS Care* **2020**, *32*, 50-56, doi:10.1080/09540121.2019.1653440.
17. Dodge, B.; Van Der Pol, B.; Rosenberger, J.G.; Reece, M.; Roth, A.M.; Herbenick, D.; Fortenberry, J.D. Field collection of rectal samples for sexually transmitted infection diagnostics among men who have sex with men. *Int J STD AIDS* **2010**, *21*, 260-264, doi:10.1258/ijsa.2009.009056.
18. Eaton, E.F.; Austin, E.L.; Dodson, C.K.; Heudebert, J.P.; Jackson, D.; Muzny, C.A. Do young black men who have sex with men in the deep south prefer traditional over alternative STI testing? *PLoS One* **2018**, *13*, e0209666, doi:10.1371/journal.pone.0209666.
19. Feinstein, B.A.; Dellucci, T.V.; Graham, S.; Parsons, J.T.; Mustanski, B. Sexually transmitted infections among young men who have sex with men: Experiences with diagnosis, treatment, and reinfection. *Sex Res Social Policy* **2018**, *15*, 172-182, doi:10.1007/s13178-017-0312-y.
20. Fields, E.L.; Long, A.; Dangerfield, D.T., 2nd; Morgan, A.; Uzzi, M.; Arrington-Sanders, R.; Jennings, J.M. There's an App for That: Using Geosocial Networking Apps to Access Young Black Gay, Bisexual, and other MSM at Risk for HIV. *Am J Health Promot* **2020**, *34*, 42-51, doi:10.1177/0890117119865112.
21. Flowers, P.; Riddell, J.; Park, C.; Ahmed, B.; Young, I.; Frankis, J.; Davis, M.; Gilbert, M.; Estcourt, C.; Wallace, L., et al. Preparedness for use of the rapid result HIV self-test by gay men and other men who have sex with men (MSM): a mixed methods exploratory study among MSM and those involved in HIV prevention and care. *HIV Med* **2017**, *18*, 245-255, doi:10.1111/hiv.12420.
22. Frye, V.; Wilton, L.; Hirshfield, S.; Chiasson, M.A.; Lucy, D.; Usher, D.; McCrossin, J.; Greene, E.; Koblin, B. Preferences for HIV test characteristics among young, Black Men Who Have Sex With Men (MSM) and transgender women: Implications for consistent HIV testing. *PLoS One* **2018**, *13*, e0192936, doi:10.1371/journal.pone.0192936.
23. Gilbert, M.; Hottes, T.S.; Kerr, T.; Taylor, D.; Fairley, C.K.; Lester, R.; Wong, T.; Trussler, T.; Marchand, R.; Shoveller, J., et al. Factors associated with intention to use internet-based testing for sexually transmitted infections among men who have sex with men. *J Med Internet Res* **2013**, *15*, e254, doi:10.2196/jmir.2888.
24. Gilbert, M.; Thomson, K.; Salway, T.; Haag, D.; Grennan, T.; Fairley, C.K.; Buchner, C.; Krajden, M.; Kendall, P.; Shoveller, J., et al. Differences in experiences of barriers to STI testing between clients of the internet-based diagnostic testing service GetCheckedOnline.com and an STI clinic in Vancouver, Canada. *Sex Transm Infect* **2019**, *95*, 151-156, doi:10.1136/sextrans-2017-053325.
25. Gu, J.; Lau, J.T.; Tsui, H. Psychological factors in association with uptake of voluntary counselling and testing for HIV among men who have sex with men in Hong Kong. *Public Health* **2011**, *125*, 275-282, doi:10.1016/j.puhe.2011.01.010.

26. Heijman, T.; Zuure, F.; Stolte, I.; Davidovich, U. Motives and barriers to safer sex and regular STI testing among MSM soon after HIV diagnosis. *BMC Infect Dis* **2017**, *17*, 194, doi:10.1186/s12879-017-2277-0.
27. Holt, M.; Bernard, D.; Race, K. Gay men's perceptions of sexually transmissible infections and their experiences of diagnosis: 'part of the way of life' to feeling 'dirty and ashamed'. *Sex Health* **2010**, *7*, 411-416, doi:10.1071/sh09117.
28. Hottes, T.S.; Farrell, J.; Bondyra, M.; Haag, D.; Shoveller, J.; Gilbert, M. Internet-based HIV and sexually transmitted infection testing in British Columbia, Canada: opinions and expectations of prospective clients. *J Med Internet Res* **2012**, *14*, e41, doi:10.2196/jmir.1948.
29. Hoyos, J.; Belza, M.J.; Fernández-Balbuena, S.; Rosales-Statkus, M.E.; Pulido, J.; de la Fuente, L. Preferred HIV testing services and programme characteristics among clients of a rapid HIV testing programme. *BMC Public Health* **2013**, *13*, 791, doi:10.1186/1471-2458-13-791.
30. Hoyos, J.; Maté, T.; Guerras, J.M.; Donat, M.; Agustí, C.; Kuske, M.; Fuertes, R.; Chanos, S.; Pichon, F.; Sordo, L., et al. Preference towards HIV Self-Testing above Other Testing Options in a Sample of Men Who Have Sex with Men from Five European Countries. *Int J Environ Res Public Health* **2021**, *18*, doi:10.3390/ijerph18094804.
31. Iribarren, S.; Lentz, C.; Sheinfil, A.Z.; Giguere, R.; Lopez-Rios, J.; Dolezal, C.; Frasca, T.; Balán, I.C.; Tagliaferri Rael, C.; Brown, W., 3rd, et al. Using an HIV Self-test Kit to Test a Partner: Attitudes and Preferences Among High-Risk Populations. *AIDS Behav* **2020**, *24*, 3232-3243, doi:10.1007/s10461-020-02885-3.
32. Jamil, M.S.; Prestage, G.; Fairley, C.K.; Grulich, A.E.; Smith, K.S.; Chen, M.; Holt, M.; McNulty, A.M.; Bavinton, B.R.; Conway, D.P., et al. Effect of availability of HIV self-testing on HIV testing frequency in gay and bisexual men at high risk of infection (FORTH): a waiting-list randomised controlled trial. *Lancet HIV* **2017**, *4*, e241-e250, doi:10.1016/s2352-3018(17)30023-1.
33. Katz, D.A.; Golden, M.R.; Hughes, J.P.; Farquhar, C.; Stekler, J.D. HIV Self-Testing Increases HIV Testing Frequency in High-Risk Men Who Have Sex With Men: A Randomized Controlled Trial. *J Acquir Immune Defic Syndr* **2018**, *78*, 505-512, doi:10.1097/qai.0000000000001709.
34. Knight, R.E.; Chabot, C.; Carson, A.; Thomson, K.; Haag, D.; Gilbert, M.; Shoveller, J. Qualitative analysis of the experiences of gay, bisexual and other men who have sex with men who use GetCheckedOnline.com: a comprehensive internet-based diagnostic service for HIV and other STIs. *Sex Transm Infect* **2019**, *95*, 145-150, doi:10.1136/sextrans-2018-053645.
35. Knight, V.; Wand, H.; Gray, J.; Keen, P.; McNulty, A.; Guy, R. Implementation and Operational Research: Convenient HIV Testing Service Models Are Attracting Previously Untested Gay and Bisexual Men: A Cross-sectional Study. *J Acquir Immune Defic Syndr* **2015**, *69*, e147-155, doi:10.1097/qai.0000000000000688.
36. Knussen, C.; Bingham, D.; Flowers, P. The acceptability of health service and community-based venues for syphilis testing amongst men who have sex with men: the views of potential service users in Scotland. *Public Health* **2008**, *122*, 959-961, doi:10.1016/j.puhe.2007.12.006.
37. Lea, T.; Anning, M.; Wagner, S.; Owen, L.; Howes, F.; Holt, M. Barriers to accessing HIV and sexual health services among gay men in Tasmania, Australia. *Journal of Gay & Lesbian Social Services* **2019**, *31*, 153-165, doi:10.1080/10538720.2019.1567427.
38. Lechuga, J.; Owczarzak, J.T.; Petroll, A.E. Marketing the HIV test to MSM: ethnic differences in preferred venues and sources. *Health Promot Pract* **2013**, *14*, 433-440, doi:10.1177/1524839912460870.
39. Lee, D.; Fairley, C.; Cummings, R.; Bush, M.; Read, T.; Chen, M. Men who have sex with men prefer rapid testing for syphilis and may test more frequently using it. *Sex Transm Dis* **2010**, *37*, 557-558, doi:10.1097/olq.0b013e3181d707de.
40. Leitinger, D.; Ryan, K.E.; Brown, G.; Pedrana, A.; Wilkinson, A.L.; Ryan, C.; Hellard, M.; Stoové, M. Acceptability and HIV Prevention Benefits of a Peer-Based Model of Rapid Point of Care HIV Testing for Australian Gay, Bisexual and Other Men Who Have Sex with Men. *AIDS Behav* **2018**, *22*, 178-189, doi:10.1007/s10461-017-1888-1.
41. Llewellyn, C.; Pollard, A.; Smith, H.; Fisher, M. Are home sampling kits for sexually transmitted infections acceptable among men who have sex with men? *J Health Serv Res Policy* **2009**, *14*, 35-43, doi:10.1258/jhsrp.2008.007065.
42. Martin, L.; Knight, V.; Ryder, N.; Lu, H.; Read, P.J.; McNulty, A. Client feedback and satisfaction with an express sexually transmissible infection screening service at an inner-city sexual health center. *Sex Transm Dis* **2013**, *40*, 70-74, doi:10.1097/OLQ.0b013e318275343b.

43. Martin, L.; Knight, V.; Read, P.J.; McNulty, A. Clients' preferred methods of obtaining sexually transmissable infection or HIV results from Sydney Sexual Health Centre. *Sex Health* **2013**, *10*, 91-92, doi:10.1071/sh12062.
44. Maxwell, S. General Practitioners' views and experiences on the barriers and facilitators that men who have sex with men have when accessing primary care for HIV testing and sexual health screening. *Prim Health Care Res Dev* **2018**, *19*, 205-209, doi:10.1017/s1463423617000627.
45. Medline, A.; Daniels, J.; Marlin, R.; Young, S.; Wilson, G.; Huang, E.; Klausner, J.D. HIV Testing Preferences Among MSM Members of an LGBT Community Organization in Los Angeles. *J Assoc Nurses AIDS Care* **2017**, *28*, 363-371, doi:10.1016/j.jana.2017.01.001.
46. Miners, A.; Nadarzynski, T.; Witzel, C.; Phillips, A.N.; Cambiano, V.; Rodger, A.J.; Llewellyn, C.D. Preferences for HIV testing services among men who have sex with men in the UK: A discrete choice experiment. *PLoS Med* **2019**, *16*, e1002779, doi:10.1371/journal.pmed.1002779.
47. Pant Pai, N.; Smallwood, M.; Desjardins, L.; Goyette, A.; Birkas, K.G.; Vassal, A.F.; Joseph, L.; Thomas, R. An Unsupervised Smart App-Optimized HIV Self-Testing Program in Montreal, Canada: Cross-Sectional Study. *J Med Internet Res* **2018**, *20*, e10258, doi:10.2196/10258.
48. Pollard, A.; Llewellyn, C.; Smith, H.; Richardson, D.; Fisher, M. Opt-out testing for HIV: perspectives from a high prevalence community in south-east England, UK. *Int J STD AIDS* **2013**, *24*, 307-312, doi:10.1177/0956462412472424.
49. Ryan, K.E.; Pedrana, A.; Leitinger, D.; Wilkinson, A.L.; Locke, P.; Hellard, M.E.; Stoové, M. Trial and error: evaluating and refining a community model of HIV testing in Australia. *BMC Health Serv Res* **2017**, *17*, 692, doi:10.1186/s12913-017-2635-z.
50. Scheim, A.I.; Travers, R. Barriers and facilitators to HIV and sexually transmitted infections testing for gay, bisexual, and other transgender men who have sex with men. *AIDS Care* **2017**, *29*, 990-995, doi:10.1080/09540121.2016.1271937.
51. Skolnik, H.S.; Phillips, K.A.; Binson, D.; Dilley, J.W. Deciding where and how to be tested for HIV: what matters most? *J Acquir Immune Defic Syndr* **2001**, *27*, 292-300, doi:10.1097/00126334-200107010-00013.
52. Spielberg, F.; Branson, B.M.; Goldbaum, G.M.; Lockhart, D.; Kurth, A.; Celum, C.L.; Rossini, A.; Critchlow, C.W.; Wood, R.W. Overcoming barriers to HIV testing: preferences for new strategies among clients of a needle exchange, a sexually transmitted disease clinic, and sex venues for men who have sex with men. *J Acquir Immune Defic Syndr* **2003**, *32*, 318-327, doi:10.1097/00126334-200303010-00012.
53. Strömdahl, S.; Liljeros, F.; Thorson, A.E.; Persson, K.I.; Forsberg, B.C. HIV testing and prevention among foreign-born Men Who have Sex with Men: an online survey from Sweden. *BMC Public Health* **2017**, *17*, 139, doi:10.1186/s12889-016-3992-y.
54. Sun, C.J.; Stowers, J.; Miller, C.; Bachmann, L.H.; Rhodes, S.D. Acceptability and feasibility of using established geosocial and sexual networking mobile applications to promote HIV and STD testing among men who have sex with men. *AIDS Behav* **2015**, *19*, 543-552, doi:10.1007/s10461-014-0942-5.
55. Tobin, K.; Edwards, C.; Flath, N.; Lee, A.; Tormohlen, K.; Gaydos, C.A. Acceptability and feasibility of a Peer Mentor program to train young Black men who have sex with men to promote HIV and STI home-testing to their social network members. *AIDS Care* **2018**, *30*, 896-902, doi:10.1080/09540121.2018.1442553.
56. Wayal, S.; Llewellyn, C.; Smith, H.; Fisher, M. Home sampling kits for sexually transmitted infections: preferences and concerns of men who have sex with men. *Cult Health Sex* **2011**, *13*, 343-353, doi:10.1080/13691058.2010.535018.
57. Witzel, T.C.; Bourne, A.; Burns, F.M.; Rodger, A.J.; McCabe, L.; Gabriel, M.M.; Gafos, M.; Ward, D.; Collaco-Moraes, Y.; Dunn, D.T., et al. HIV self-testing intervention experiences and kit usability: results from a qualitative study among men who have sex with men in the SELPHI (Self-Testing Public Health Intervention) randomized controlled trial in England and Wales. *HIV Med* **2020**, *21*, 189-197, doi:10.1111/hiv.12818.
58. Witzel, T.C.; Melendez-Torres, G.J.; Hickson, F.; Weatherburn, P. HIV testing history and preferences for future tests among gay men, bisexual men and other MSM in England: results from a cross-sectional study. *BMJ Open* **2016**, *6*, e011372, doi:10.1136/bmjopen-2016-011372.
59. Witzel, T.C.; Rodger, A.J.; Burns, F.M.; Rhodes, T.; Weatherburn, P. HIV Self-Testing among Men Who Have Sex with Men (MSM) in the UK: A Qualitative Study of Barriers and Facilitators, Intervention Preferences and Perceived Impacts. *PLoS One* **2016**, *11*, e0162713, doi:10.1371/journal.pone.0162713.
60. Wohlfeiler, D.; Hecht, J.; Volk, J.; Fisher Raymond, H.; Kennedy, T.; McFarland, W. How can we improve online HIV and STD prevention for men who have sex with men? Perspectives of hook-up

website owners, website users, and HIV/STD directors. *AIDS Behav* **2013**, *17*, 3024-3033, doi:10.1007/s10461-012-0375-y.

61. Wray, T.B.; Chan, P.A.; Simpanen, E.; Operario, D. A Pilot, Randomized Controlled Trial of HIV Self-Testing and Real-Time Post-Test Counseling/Referral on Screening and Preventative Care Among Men Who Have Sex with Men. *AIDS Patient Care STDS* **2018**, *32*, 360-367, doi:10.1089/apc.2018.0049.
